# Environmental Changes and the Impact on the Human Infections by Dengue, Chikungunya and Zika Viruses in Northern Brazil, 2010–2019

**DOI:** 10.3390/ijerph191912665

**Published:** 2022-10-03

**Authors:** Robson dos Santos Souza Marinho, Rodrigo Lopes Sanz Duro, Mânlio Tasso de Oliveira Mota, James Hunter, Ricardo Sobhie Diaz, Fernando Shinji Kawakubo, Shirley Vasconcelos Komninakis

**Affiliations:** 1 Retrovirology Laboratory, Federal University of São Paulo, São Paulo 04039-032, Brazil; 2 Faculty of Philosophy, Letters and Human Sciences, University of São Paulo, São Paulo 05508-000, Brazil

**Keywords:** environmental change, arbovirus infections, human, Brazil

## Abstract

Environmental changes are among the main factors that contribute to the emergence or re-emergence of viruses of public health importance. Here, we show the impact of environmental modifications on cases of infections by the dengue, chikungunya and Zika viruses in humans in the state of Tocantins, Brazil, between the years 2010 and 2019. We conducted a descriptive and principal component analysis (PCA) to explore the main trends in environmental modifications and in the cases of human infections caused by these arboviruses in Tocantins. Our analysis demonstrated that the occurrence of El Niño, deforestation in the Cerrado and maximum temperatures had correlations with the cases of infections by the Zika virus between 2014 and 2016. El Niño, followed by La Niña, a gradual increase in precipitation and the maximum temperature observed between 2015 and 2017 were shown to have contributed to the infections by the chikungunya virus. La Niña and precipitation were associated with infections by the dengue virus between 2010 and 2012 and El Niño contributed to the 2019 outbreak observed within the state. By PCA, deforestation, temperatures and El Niño were the most important variables related to cases of dengue in humans. We conclude from this analysis that environmental changes (deforestation and climate change) presented a strong influence on the human infections caused by the dengue, chikungunya and Zika viruses in Tocantins from 2010 to 2019.

## 1. Introduction

Environmental changes have significantly contributed to the emergence of infectious diseases causing major impacts on public health worldwide. Most of these infections are viral in origin with their emergence or re-emergence triggered by human activities or natural phenomena that modify the environment, favoring transmission [1]. 

Deforestation represents one of the main causes of land use change throughout the world. Its main causes are commercial agriculture, livestock, urbanization, fires and illegal logging [2]. It is estimated that commercial agriculture is responsible for 70% of forest destruction in tropical and subtropical countries [3]. According to the data of the Global Forest Watch [4], Brazil lost 78% of tree cover between 2001 and 2018 and 66% of this loss was due to urbanization and commercial agriculture.

Climate change can be understood as any change in climate over the years. The events El Niño/La Niña modulate the climate in tropical regions; in years of the occurrence of El Niño, the average daily maximum temperature tends to increase, and the dry period is longer. On the other hand, in years of La Niña, the average temperature tends to decrease. Accompanying the drop in temperature and concomitant greater precipitation (rainfall), there are more frequent episodes of flooding, especially in urban areas [5]. In Brazil, some climate change scenarios involving the El Niño/La Niña natural events have been reported [6]. According to the National Oceanic and Atmospheric Administration [7], occurrences of extreme El Niño and La Niña events were registered between the years 2010 and 2019. These can directly impact the density and spread of mosquitoes and arboviruses [8]. 

It is known that the transmission dynamics of arboviruses in tropical regions is multifactorial involving many aspects including environmental factors related to deforestation and climatic conditions [9]. Vasconcelos and Calisher [10] studied the emergence of human arboviral diseases in the Americas from 2000 to 2016. They showed that these environmental changes are among the principal causes of arbovirus outbreaks. 

Mosquitoes are hematophagous vectors for many pathogenic viruses of public health importance. In nature, mosquito-borne viruses maintain a life cycle between mosquitoes and vertebrate animals [11]. Viruses propagate extensively in the tissues of mosquitoes and these mosquitoes become a reservoir of viruses and are able to transmit them to a vertebrate host through the next blood meal [12]. To survive and circulate efficiently between two distinct host environments, mosquito-borne viruses have developed delicate and intelligent strategies to comprehensively exploit host and vector factors [13].

The favorable conditions for *Aedes aegypti* and *Aedes albopictus* breeding contribute to the spread and outbreaks of other diseases caused by arboviruses and that infect this same vector, maintaining local outbreaks and epidemics [14]. The high diversity of arboviruses found in Brazil, the environmental plasticity of the *Aedes* spp. Vector, environmental changes and the growing proximity of humans in forest regions can lead to new outbreaks and epidemics of new arboviruses circulating in the country, but which still form discreetly, as is the case with Oropouche and Mayaro [15]. This reinforces the importance of the vector control of these arboviruses. 

Dengue (DENV), chikungunya (CHIKV) and Zika (ZIKV) viruses are endemic arboviruses in Brazil. The first dengue epidemic occurred in 1981; subsequently, outbreaks were reported in all regions of the country. In 2014 and 2015, CHIKV and ZIKV, respectively, were introduced into the country causing epidemics in many Brazilian states [16]. These viruses are transmitted to humans mainly by mosquitoes of the genus Aedes; however, other forms of ZIKV transmission have also been described [17].

Some studies have documented the circulation and infections by DENV, CHIKV and ZIKV in Tocantins. Bezerra et al. [18], in a systematic review showing the entry of DENV serotypes and their geographic distribution in Brazilian states, revealed that the virus has circulated in Tocantins with the introduction of serotype 2 in 1991. Subsequently, the serotypes 3, 1 and 4 were detected in the state in 1999, 2000 and 2011, respectively. In a study conducted by Milagres et al. [19], the co-circulation of these three arboviruses during an outbreak of ZIKV between 2015 and 2016 was demonstrated. Another study that used molecular methods to detect coinfection with CHIKV and DENV serotype 2 in serum samples of patients, also proved the circulation of these viruses in the state [20].

Although molecular epidemiology studies of DENV, CHIKV and ZIKV in Tocantins are reported in the literature, the available information on the influence of environmental modifications on the outbreaks of these arboviruses in the state is still scarce. We believe that environmental changes altered the cases of DENV, ZIKV and CHIKV infections in humans in the state of Tocantins between the years 2010 and 2019. Therefore, the objective of our work was to show the impact of environmental modifications on the cases of infections by DENV, CHIKV and ZIKV in humans in the state of Tocantins, Brazil, between the years 2010 and 2019.

## 2. Materials and Methods

### 2.1. Study Area 

**Figure 1:**
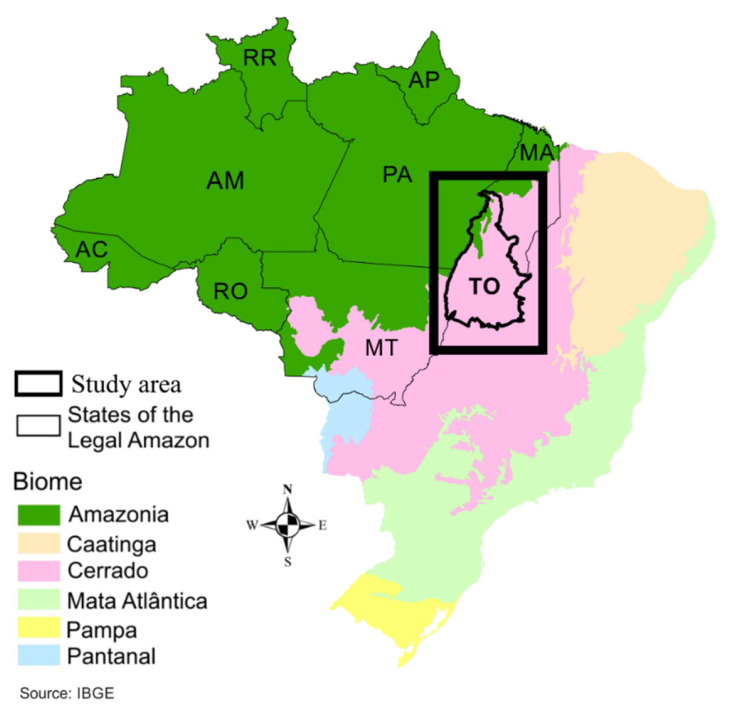
Location of the study area. This map was built using the software ArcGIS. AC: Acre, AM: Amazônia, AP: Amapá, MA: Maranhão, MT: Mato Grosso, PA: Pará, RR: Roraima, RO: Rondônia, TO: Tocantins.

Tocantins is a Brazilian state belonging to the northern region of the country. It comprises an area of 277,720.520 km^2^ and is located in an area of transition between the Cerrado and Amazon biomes, which occupy approximately 91% and 9% of the state territory, respectively. It borders the states of Goiás (South), Piaui (East), Maranhão (Northeast), Bahia (Southeast), Pará (Northwest) and Mato Grosso (Southwest) (Figure 1).

According to the Brazilian Institute of Geography and Statistics (IBGE), the state is the fourth most populous in the Northern Region with an estimated population of 1,555,229 million inhabitants and a demographic composition of 79% urban and 31% rural residents. In 2010, the state had a human development index (HDI) of 0.699, third highest among the states of the North Region of Brazil, and fourteenth among the other states in the country [21].

### 2.2. Study Design

This is an observational study that uses environmental data and data of infections by DENV, CHIKV and ZIKV in humans that occurred in the state of Tocantins between the years 2010–2019. To show a relation between the environmental conditions and the cases of human infections by these arboviruses, we examined seven variables: deforestation in the Cerrado (“Cerrado Defor”, km^2^) and in the Amazon rainforest (“Amazon rainforest Defor”, km^2^), occurrence and intensity of El Niño and La Niña (“ONI”), average annual maximum temperature (“Max. Temp”, °C), average annual minimum temperature (“Min. Temp”, °C) and annual precipitation (“precipitation”, mm). To determine the environmental variables that had the greatest influence on the cases of infections by DENV in humans in these ten years, we applied a principal component analysis (PCA), as previously described by Lorenz et al. [22], with some modifications. All data collected in this study are publicly available on online platforms. 

### 2.3. Annual Accumulated Deforestation

The data for shallow cut deforestation between the years 2010 and 2019 in the state of Tocantins were obtained from the National Institute for Space Research (INPE) using publicly available information from PRODES (Monitoring the Brazilian Amazon Forest by Satellite) on the TerraBrasilis web site (http://terrabrasilis.dpi.inpe.br, accessed on 22 September 2020). We determined the rates of annual deforestation in the two biomes present in the state (Cerrado and Amazon Rainforest). The PRODES data were used due to their reliability for identifying deforestation patches [23].

### 2.4. Occurrence and Intensity of El Niño and La Niña

The Oceanic Niño Index (ONI) is defined as the consecutive three month average equatorial Sea Surface Temperature (SST) departures in the Niño 3.4 region. This index helps to put events of occurrence and intensity of El Niño and La Niña in a historical perspective characterizing them with a base in its values. In this way, El Niño is characterized by an ONI value greater than or equal to +0.5 °C and La Niña by an ONI value less or equal to −0.5 °C. By historical standards, to be classified as a full episode of El Niño or La Niña, these limits must be exceeded for a period of at least 5 consecutive seasons of 3 months overlapping. The historical episodes of El Niño and La Niña based on the ONI are calculated using Extended Reconstructed Sea Surface Temperature (ERSST) v5. The data related to occurrences of El Niño and La Niña phenomena and their respective intensities were acquired from the website of the National Oceanic and Atmospheric Administration (NOAA). (https://www.cpc.ncep.noaa.gov/products/precip/CWlink/MJO/enso.shtml, accessed on 30 November 2020).

### 2.5. Annual Maximum and Minimum Temperature and Precipitations

We obtained the historical data on temperature and precipitation from the National Institute of Meteorology (https://portal.inmet.gov.br/dadoshistoricos, accessed on 2 September 2020). We calculated the averages of maximum and minimum temperatures and precipitation for each of the studied years. 

### 2.6. Register of Cases of Probable and Confirmed Dengue, Chikungunya and Zika in Tocantins

A database was created with the averages of the numbers of cases by years of occurrence for these arboviruses based on information from the Secretary of Health of Tocantins (https://central3.to.gov.br/arquivo/492214/, accessed on 22 September 2020). The analysis included all probable (clinically diagnosed) and confirmed (serological and molecular) cases of people with the disease onset during this period established for analysis. The numbers of cases analyzed were from the entire state.

### 2.7. Statistical Analysis

Initially, we performed a descriptive analysis to explore and show the main trends. In this analysis, we observed the most recurrent environmental changes within these ten years (2010–2019) and established a relationship with the increase in the number of DENV, ZIKV and CHIKV cases. Posteriorly, a principal components analysis was used to show the environmental variables that had the greatest influence on the cases of infection by DENV in humans. It was not possible to apply this analysis to the cases of CHIKV and ZIKV due to limited availability of data. The PCA generates two factors, the first of which represents the main component (dimension 1 or Dim.1). The second factor is orthogonal to the first, corresponding (dimension 2 or Dim.2). Each dimension/component is the result of individual contributions of each of the original variables. Although PCA as a technique can include multiple dimensions, our model focuses on just the first two. These two dimensions have a set of eigenvectors. Each presents an eigenvalue that together explain the contribution of the total variation in the data. The PCA was based on a correlation matrix that was performed after standardization of the Z score. All analyses were performed in R software version 4.1.0.

## 3. Results

### 3.1. Deforestation

**Figure 2:**
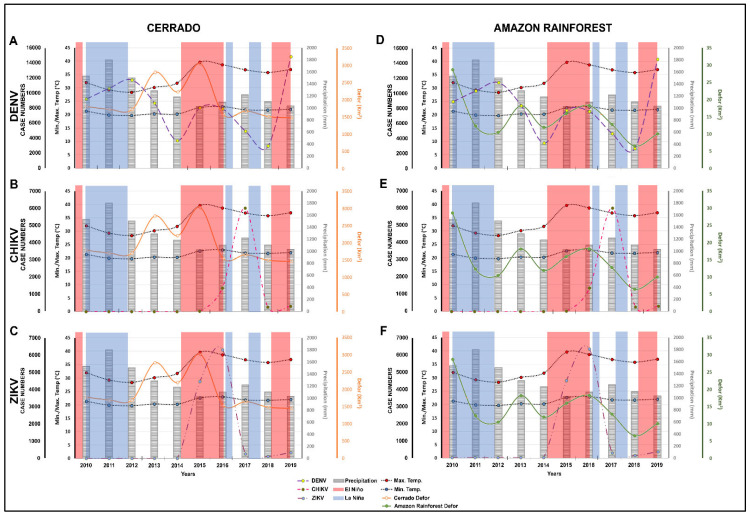
Distribution of the environmental modifications and of the cases of human infections by the DENV, CHIKV and ZIKV between the years 2010 and 2019 in Tocantins, Brazil. (**A**–**C**) Show the cases of human infections by dengue, chikungunya and Zika viruses, respectively, and their relationships with the natural phenomena (temperatures, precipitation, El Niño/La Niña) and deforestation that occurred in the Cerrado biome within Tocantins from 2010 to 2019. (**D**–**F**) Show the same cases of human infections by dengue, chikungunya and Zika viruses, respectively, and their relationships with changes in temperature, precipitation, El Niño/La Niña and the deforestation that occurred in the Amazon Rainforest biome within Tocantins from 2010 to 2019.

The analyses of deforestation that occurred from 2010 to 2019 in Tocantins showed a large occurrence of these practices in this period. According to TerraBrasilis (PRODES, INPE), the portion of the Amazon rainforest in the state showed greater deforestation in 2010 with 28.93 km^2^ and the portion of the Cerrado biome had the largest area devastated in 2015, corresponding to 3063.38 km^2^, as shown in the Supplementary Materials (Table S1). The years 2010 and 2015 were critical for deforestation in Tocantins, with regards to 2010 in the Amazon rainforest and 2015 in the Cerrado, as shown in Figure 2.

### 3.2. El Niño and La Niña

In this period, we observed three episodes of El Niño. In 2010, the overlapping analysis of 3 months from DJF (December, January, February), JFM (January, February, March) and FMA (February, March, April) showed the ONI with values of 1.5, 1.3 and 0.9, respectively. We also observed a gradual increase in the intensity of the occurrence of El Niño in the period of overlapping analysis of 3 months from OND (October, November, December) 2014 to NDJ (November, December, January) 2015 with the ONI varying from 0.6 to 2.6, which was the longest occurrence of this phenomenon. It can be also noted that in 2016 there was a continuity of the phenomenon with a reduction in its intensity; ONI values decreasing from 2.5 in DJF to 0.5 in AMJ (April, May, June) in this year were identified. Another episode of El Niño was observed from SON (September, October, November) 2018 to MJJ (May, June, July) 2019. This then continued through OND and NDJ of that year with the ONI varying from 0.5 to 0.9. 

Some occurrences of La Niña were also observed. From the period of MJJ 2010 to FMA 2012, an intense occurrence of La Niña was observed with the ONI ranging from −0.5 to −1.7. This was the longest period of this phenomenon within the time frame of interest. Starting with the second semester of 2016, JAS (July, August, September) to NDJ, which had an ONI of −0.6 and −0.7, we again observed the occurrence of La Niña from SON of 2017 to FMA of 2018 with an ONI ranging from −0.6 to −1.0. In Figure 2, we show the occurrences of El Niño/La Niña during this period; the respective ONI values are indicated in the Supplementary Materials (Table S2).

### 3.3. Temperatures and Precipitation

The analysis of the maximum and minimum temperatures from 2010 to 2019 in Tocantins showed that 2015–2016 were the crucial years for climate change as in those years, the state started to register higher maximum temperature levels than before. In contrast, we note that the lowest minimum temperatures recorded were during the years 2010 to 2014. Regarding the annual precipitation rates, we observed that between the years 2010 and 2012, the state had the highest rain indices in relation to the other years analyzed, as shown in the Supplementary Materials (Table S3) and Figure 2. 

### 3.4. Cases of Dengue, Chikungunya and Zika

For DENV, we verified that there was a gradual increase in the number of cases from 2010 to 2012 with rates of increase of 9.62%, 13.52% and 55.15%, respectively. Between 2013 and 2014, there was a drop in the number of registered cases. However, in 2015 and 2016, a further increase in the number of cases was observed (23.65% and 8.78%, respectively). In 2017 and 2018, a decrease in cases was recorded, which lasted until 2019 when the state faced an outbreak of this virus. Tocantins led the national ranking with the highest prevalence (79.60%) of dengue cases in Brazil. For CHIKV and ZIKV, it was observed that the state started to register and to disclose the circulation of these viruses in 2015. The state had the greatest increase in the number of cases registered of CHIKV in 2017 with 77.06% and 2016 was the crucial year for ZIKV infections with a prevalence of 95.87%. The Secretary of Health of Tocantins reported outbreaks of ZIKV in 2016 and CHIKV in 2017 in all regions of the state, as shown in the Supplementary Materials (Table S4) and Figure 2.

### 3.5. Distribution of the Environmental Modifications and Arbovirus Cases between 2010 and 2019

We observed that there was a peak of CHIKV and ZIKV cases in Tocantins between the years 2015 and 2017 and associated with this we noted the occurrence of the 2015/16 super El Niño and an increase in temperature and precipitation in the state. We also noted that 2010, 2011, 2012 and 2019 were the years with the highest number of cases of DENV infection in Tocantins. We identified that 2010–2012 were the years in which the highest precipitation rates were recorded in the state. During this period, there was the highest occurrence of La Niña. Figure 2 illustrates the distribution of environmental changes and arbovirus cases between 2010 and 2019 in Tocantins.

### 3.6. Principal Component Analysis (PCA)

**Figure 3:**
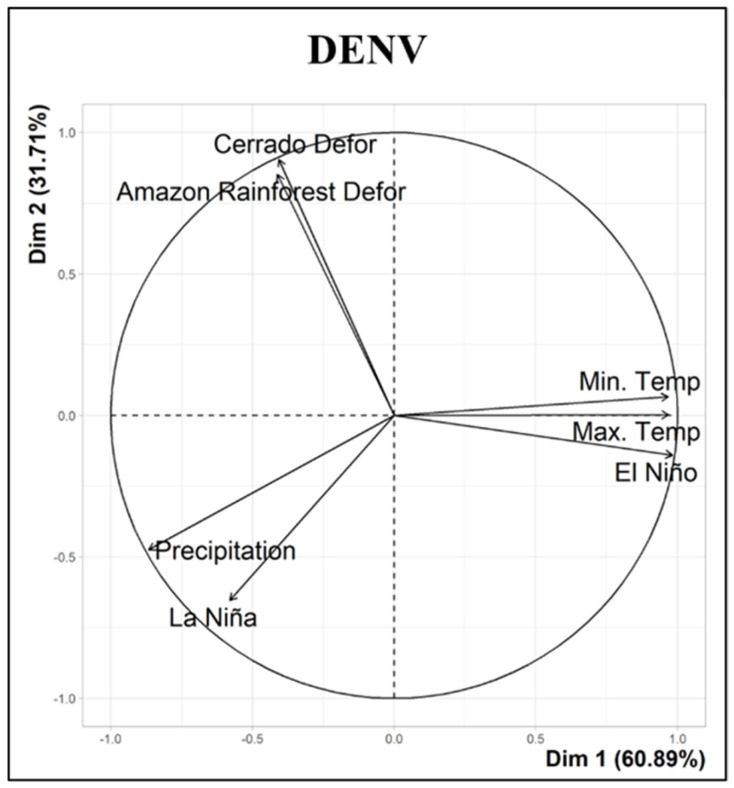
The most influential environmental variables. PCA for DENV showing the most influential environmental variables.

Through the PCA analysis, it was possible to note the variables with the greatest influence for DENV. The first two components (Dim 1 and Dim 2) explained 92.60% of the variance in the model (Figure 3). 

The most important variables with respect to DENV cases were deforestation (Cerrado and Amazon rainforest), temperatures (maximum and minimum) and El Niño, which had strongest associations with the increase in the number of DENV cases registered by the Secretary of Health of Tocantins during the analyzed period. Details are shown in Table 1.

## 4. Discussion

In this study, we showed that the occurrences of DENV, CHIKV and ZIKV cases in humans in the state of Tocantins, Brazil, in the period 2010–2019 presented correlations with the environmental conditions. Of the environmental aspects analyzed in this research, some showed a strong influence on the cases of human infections by these three arboviruses. There are few studies that demonstrate the correlation between environmental factors and the incidence of arboviral diseases in Brazil. Thus, the aim of our work was to contribute to the understanding of the causes that influence the circulation, spread and maintenance of arboviruses in Brazil. 

Despite this study focusing only on the environmental variables, socio-economic variables also have a huge impact on the circulation of arboviruses in human populations. Trigueiro et al. [24] discussed the spatial variability of the recent causes of deforestation in the Brazilian Cerrado. The authors stressed that deforestation in this biome has increased considerably, reaching higher rates than in the Amazon region. Recent studies show that agriculture and livestock are the main causes of deforestation in this biome [25,26,27,28]. The increase in non-vegetated areas in Tocantins in these last ten years has been an important trend shown by the Brazil MapBiomas platform, with native vegetation being replaced mainly by areas of agriculture, livestock and urban infrastructure (MapBiomas, 2021). Furthermore, according to a report generated by Chain Reaction Research [29], soy plays a key role in the economy of Tocantins and is the most important agricultural production in the state. Additionally, a recent study showed that social exclusion can alter the way in which the climate and deforestation impact the circulation of Leishmaniasis, a vector-borne disease the circulation of which is affected by ecological variables [30].

Another important analytic result in our work was found by specifying the frequency and intensity of the natural phenomena El Niño and La Niña. Chen et al. [31] sought to understand the mechanism that formed the super El Niño of 2015/16 and showed that it was ranked as the third most intense since 1950 and resulted in a major impact on the Earth’s natural climate system. This super El Niño began to develop at the end of 2014, gained strength throughout 2015 and only decreased in intensity in 2016 [7]. Furthermore, Luo et al. [32], studying the inter-basin sources for two year predictability of the multiannual event La Niña in 2010–2012, reported that this was one of the largest intensities of this event. It caused intense rainfall in many regions of the world inducing floods and a cold and humid climate. 

Analyzing the maximum and minimum temperatures as well as the precipitation rates in Tocantins, we can verify that there is a relation with the occurrences of El Niño and La Niña. Some studies show that El Niño causes warming and La Niña cooling and that this can change the average temperature in many countries, including Brazil [33,34,35]. We observed that in 2015 and 2016, the state of Tocantins reached higher maximum temperatures during an intense El Niño. According to the European Center for Medium-Range Weather Forecasts [36], the 2015/16 El Niño significantly contributed to record global temperatures. Moreover, in our analyses, we observed that from 2010 to 2012, Tocantins recorded higher rainfall and lower minimum temperatures, which compares with the worldwide observation of the influence of La Niña on world temperature. A study conducted by Moura et al. [37] showed the relationship of the El Niño and La Niña phenomena with precipitation, evapotranspiration and temperature in the Amazon basin. The authors showed that La Niña increases precipitation rates and consequently decreases atmospheric temperature. This corroborates our data and reinforces the idea of the relationship between El Niño/La Niña and climate change.

We observe there was a peak in cases of CHIKV and ZIKV in Tocantins between the years 2015 and 2017. Additionally, we note the occurrence of the super El Niño of 2015/16 when the temperature and precipitation in the state increased significantly. Many disease outbreaks were associated with the El Niño of 2015/16; this phenomenon provoked climatic and environmental anomalies causing increases in temperatures, droughts and extreme rainfall in many regions of the world, which favored the occurrence of outbreaks of a wide range of diseases of public health concern, including chikungunya and Zika [38]. Chretien et al. [39] showed that the El Niño of 2015/16 led to optimal ecological conditions for emerging and re-emerging disease vectors that contributed to infections in susceptible humans. Therefore, in our work, we reinforce the importance of El Niño in the incidence of CHIKV and ZIKV in Brazil. 

By analyzing the PCA results, we were able to show that deforestation (Cerrado and Amazon rainforest), temperatures (maximum and minimum) and El Niño had an impact on the cases of DENV registered during the analyzed period. Kalbus et al. [40] explored the influence of deforestation in the Amazon Forest on the incidence of dengue in the state of Amazonas, Brazil, in the period 2007–2017. They showed that both the incidence of dengue and deforestation increased; in addition, the bivariate analysis performed by the authors revealed increased DENV incidences for a few years after deforestation. Our findings showed that 2010 was the year of greatest deforestation in the Amazon Forest area within the state of Tocantins. This resulted in a significant increase in cases of DENV within the state after that date. Tocantins experienced higher peaks between 2010 and 2012 and 2019. This shows that deforestation in the Amazon rainforest is one of the important factors in DENV outbreaks in Brazil. 

In addition, temperature and precipitation patterns are known to influence the seasonality of dengue transmission [41]. In our analysis, we observed that 2010, 2011, 2012 and 2019 were the years with the highest numbers of cases of DENV infection in Tocantins. We also observed that 2010–2012 were the years in which we observed the highest precipitation rates in the state. During this period, there was the highest occurrence of La Niña. According to the National Institute for Space Research [42], La Niña affects the climate in many regions in Brazil by increasing rainfall rates. It is noteworthy that through the first seven months of 2019, South America experienced a moderate El Niño. This phenomenon may have contributed to the DENV outbreak registered in that year. According to Moraes et al. [43], El Niño/La Niña and precipitation contribute to favorable climatic conditions for the spread of the mosquito vectors of DENV. This may explain the influence that these environmental conditions can have on the incidence of dengue in Tocantins. 

We note some limitations in our work due to the underreporting of cases of DENV, CHIKV and ZIKV. Even using data made available by the State Health Department, it is likely that there will be many more cases of these viruses in Tocantins. The lack of complete socio-economic data available for the entire state of Tocantins made it difficult to include socio-economic variables and other variables in our study. We also did not have access to the Rapid Index Survey for Aedes aegypti (LIRAa) to obtain entomological indicators that would allow us to find out the distribution of the vector, and it was not possible to generate a species distribution model. Furthermore, we know that in Brazil, other arboviruses circulate. These include the Mayaro, Oropouche, yellow fever, West Nile, Saint Louis, and Rocio viruses. However, it was not possible to include these viruses in our study because the information available about their circulation in Tocantins are scarce. It is also important to mention that our study did not explore in detail the question of forest degradation and its contribution to the circulation of DENV, CHIKV and ZIKV. 

This work reinforced the relationship between environmental changes and the incidence of arboviruses in Tocantins, Brazil; however, we encourage further studies in this field that involve these arboviruses and use species distribution algorithms as well as other sets of entomological and socio-economic variables. The next step in our study will be to extend this analysis to the whole of Brazil and include other endemic arboviruses.

## 5. Conclusions

The life cycle of mosquitoes is closely related to seasonal events in nature, such as El Niño, La Niña, humidity and temperature. In fact, changes in these environmental conditions can directly influence the density, dispersion and spread of mosquitoes. Added to this, human actions such as deforestation lead to an imbalance in the natural environment. We infer that deforestation and climate changes mainly influenced by the El Niño and La Niña phenomena impacted outbreaks of DENV, CHIKV and ZIKV that have occurred in the state of Tocantins between the years 2010 and 2019. In addition, the high diversity of vectors and arboviruses present in all biomes in Brazil and the rapid fragmentation and environmental destruction bring into focus the need for the monitoring of arbovirus diseases in the country. These findings suggest that the human actions and natural phenomena contribute mainly by interfering in the circulation, distribution and maintenance of their mosquito vectors. Moreover, among the environmental variables studied, deforestation is the only one induced directly by human actions, and this reinforces the importance of human awareness about the conservation of native forests for the control of arbovirus circulation in urban areas. This will contribute to the reduction in sporadic outbreaks of endemic arboviruses such as DENV, CHIKV and ZIKV.

## Figures and Tables

**Table 1:** Importance of environmental variables for the cases of DENV.

Environmental Variables	Eigenvectors ^a^	
	Dim.1	Dim.2
Amazon Rainforest Defor	0.412	0.851
Cerrado Defor	0.407	0.905
Max. Temp	0.982	0.002
Min. Temp	0.967	0.066
Precipitation	0.767	−0.141
El Niño	0.882	−0.475
La Niña	0.580	−0.354

**^a^** These are sets of values that represent the weight of each original variable on each component. Theses eigenvectors are scaled as correlation coefficients and range from +1.0 to −1.0 (passing through zero). For each component, all variables have a set of corresponding eigenvectors, and the closer to +1.0 or −1.0 the eigenvector, the more important the variable for the component is.

## Data Availability

The datasets used and analyzed during the current study are available from the corresponding author on reasonable request.

## Supplementary Materials

The following supporting information can be downloaded at: https://www.mdpi.com/article/10.3390/ijerph191912665/s1; Table S1: Deforestation in the Amazon rainforest and in the Cerrado within the state of Tocantins/Brazil from 2010 to 2019; Table S2: Occurrences and intensities of the El Niño and La Niña phenomena from 2010 to 2019; Table S3: Annual medias of maximum/minimum temperatures and precipitation from 2010 to 2019 in Tocantins/Brazil; Table S4: Number of probable and confirmed cases of infections by DENV, CHIKV and ZIKV from 2010 to 2019 in Tocantins/Brazil.
