# Supplementary material for: Environmental Changes and the Impact on the Human Infections by Dengue, Chikungunya and Zika Viruses in Northern Brazil, 2010–2019
Source: Int J Environ Res Public Health. 2022 Oct 3;19(19):12665. doi: 10.3390/ijerph191912665 (PMC9566075; doi:10.3390/ijerph191912665)
Supplement: Supplementary file 1 [file ijerph-19-12665-s001.zip › ijerph-1904862-supplementary.pdf]

Table S1: Deforestation in the Amazon rainforest and in the Cerrado within the state of Tocantins/Brazil from 2010 to 2019.

Years	Biomes / Deforested Area (Km²)	
	Amazon rainforest	Cerrado
2010	28.93	1819.80
2011	12.31	1740.80
2012	10.39	1740.80
2013	18.24	2816.82
2014	11.88	2243.35
2015	15.95	3063.38
2016	18.27	1587.19
2017	12.74	1693.45
2018	6.33	1530.09
2019	9.95	1495.61

These data were obtained of the TerraBrasilis (PRODES, INPE).

Table S2: Occurrences and intensities of the El Niño and La Niña phenomena from 2010 to 2019.

Years	Periods (months) / Oceanic Nino Index (ONI) [a]											
	DJF	JFM	FMA	MAM	AMJ	MJJ	JJA	JAS	ASO	SON	OND	NDJ
2010	1.5	1.3	0.9	0.4	-0.1	-0.6	-1.0	-1.4	-1.6	-1.7	-1.7	-1.6
2011	-1.4	-1.1	-0.8	-0.6	-0.5	-0.4	-0.5	-0.7	-0.9	-1.1	-1.1	-1.0
2012	-0.8	-0.6	-0.5	-0.4	-0.2	0.1	0.3	0.3	0.3	0.2	0.0	-0.2
2013	-0.4	-0.3	-0.2	-0.2	-0.3	-0.3	-0.4	-0.4	-0.3	-0.2	-0.2	-0.3
2014	-0.4	-0.4	-0.2	0.1	0.3	0.2	0.1	0.0	0.2	0.4	0.6	0.7
2015	0.6	0.6	0.6	0.8	1.0	1.2	1.5	1.8	2.1	2.4	2.5	2.6
2016	2.5	2.2	1.7	1.0	0.5	0.0	-0.3	-0.6	-0.7	-0.7	-0.7	-0.6
2017	-0.3	-0.1	0.1	0.3	0.4	0.4	0.2	-0.1	-0.4	-0.7	-0.9	-1.0
2018	-0.9	-0.8	-0.6	-0.4	-0.1	0.1	0.1	0.2	0.4	0.7	0.9	0.8
2019	0.8	0.8	0.8	0.8	0.6	0.5	0.3	0.1	0.1	0.3	0.5	0.5

El Niño	Neutro	La Niña

[a] Full annual episode of El Niño or La Niña with 3 months overlapping - **DJF:** December, January, February; **JFM:** January, February, March; **FMA:** February, March, April; **MAM:** March, April, May; **AMJ:** April, May, June; **MJJ:** May, June, July; **JJA:** June, July, August; **JAS:** July, August, September; **ASO:** August, September, October; **SON:** September, October, November; **OND:** October, November, December; **NDJ:** November, December, January. [b] ONI: Oceanic Niño Index in degrees Celsius. La Niña = ONI ≤ -0.5 and El niño = ONI ≥ + 0.5 ºC; ONI value between the interval of -0.4 to +0.4 corresponds to neutral, there was no occurrence of the phenomena.

These data were obtained of the National Oceanic and Atmospheric Administration (NOAA).

Table S3: Annual medias of maximum/minimum temperatures and precipitation from 2010 to 2019 in Tocantins/Brazil.

Years	Max. Temp	Min. Temp	Precipitation
2010	32.0	21.3	1529.0
2011	29.3	19.9	1798.8
2012	28.4	19.7	1500.9
2013	30.2	20.3	1289.4
2014	31.8	20.2	1187.6
2015	39.6	22.6	1024.9
2016	38.7	23.0	1100.5
2017	36.8	21.8	1220.7
2018	35.8	21.7	1103.8
2019	36.9	22.0	1031.6

These data were obtained of the National Institute of Meteorology (INMET).

Table S4: Number of probable and confirmed cases of infections by DENV, CHIKV and ZIKV from 2010 to 2019 in Tocantins/Brazil.

Years	DENV	CHIKV	ZIKV
2010	9.095	-	-
2011	10.517	-	-
2012	11.636	-	-
2013	8.564	-	-
2014	3.590	-	-
2015	7.949	29	4.472
2016	7.782	1.366	6.303
2017	4.852	6.014	272
2018	2.874	262	120
2019	14.728	317	345

These data were obtained of the Secretary of Health of the Tocantins.
